# The Registration of Qualified Nurses

**Published:** 1887-12-17

**Authors:** J. C. Steele

**Affiliations:** Superintendent of Guy's Hospital


### REGISTRATION FOR QUALIFIED NURSES.

BY J. C. STEELE, M.D., Superintendent of Guy's Hospital.

FEELING keenly the necessity of establishing a public register for nurses who have undergone hospital training and have received, or from length of service are entitled to, certificates or diplomas of competency, I have willingly acceded to the request of the council to contribute a short paper to its transactions explanatory of my views on the subject. From some facts which have come to my knowledge since, I find that the question has already been exercising the minds of many members of the Association, and that a scheme l as been already prepared, by which the important desideratum is sought to be obtained. Any remarks I may make will have but small reference to this particular scheme, the main object of the paper being rather to elicit opinion as to the best basis on which a system of nurse registration should be organised, and the advantages likely to accrue from it. Our earnest desire is to secure your aid and the aid of all interested in the "divine drudgery," to help forward a work which must sooner or later cap the efforts that have been made, and which are now in active operation, for the purpose of perfecting this important branch of women's work. The far too familiar term, "trained nurse," so constantly besetting us in our walks abroad, or at home in the advertising columns of the daily press, carries with it an assumption that the happy possessor of the title has undergone some kind of probation or apprenticeship to the art she professes, or which is professed for her by proxy. The too-credulous world is at length, although by slow degrees, becoming alive to the fact that there are nurses and nurses. We who are privileged to see much behind the scenes were aware of the paradox long ago, but it takes a long time before society at large can penetrate these mysteries. We have only to make a few inquiries into the antecedents of some members of the nursing republic to find that there are hundreds, I might with equal confidence say, thousands, and respectable women, too, who have either not had any hospital training at all, or, one of the most meagre description. Yet such claim and take a position in the commonwealth on a par with women who have probably spent some of the best years of their early life in acquiring a practical, and, in many instances, a theoretical knowledge of the art. To meet this injustice, there is a feeling abroad that the time has arrived, when the vocation of nursing may safely assert its claims to a higher life, and that its reasonable request to be recognised by the State as well as by the community as worthy of legal protection should be voluntarily conceded.

It is now pretty well known that at the chief centres of medical education in London and the provinces, there are training schools for nurses, each large hospital preferring to have its own attendants reared on the premises. Further, to extend the privileges of the hospital nursing staff to the rich, which till within the last few years had been almost entirely confined to the poor, the services of a large and increasing number of qualified nurses, recruited direct from the hospital, are utilised in nursing institutions co-operating with the hospitals. Nearly every large hospital has now such an establishment affiliated with it, and many others are contemplating its expediency. These nursing institutions, together with others under private management, absorb a very large number of women who have finished their hospital training, in London alone, probably as many as are engaged in the general hospitals. It consists also with one's experience of the fitness of things that many nurses mentally and physically prove to be better adapted for private than for hospital work; and as usually happens, their own leanings attract them in the same direction. To supply the dual demand it has become a matter of necessity that there should be a continuous stream of recruits of an eligible character ready to fill the gaps occasioned by the departures. In their initial engagement it is the custom in all the leading hospitals to require the novice to sign an undertaking giving her services for two or more years, the usual period being three years, the first year of which she acts as probationer or nurse's assistant, and the remaining two as ward, staff, or regular nurse. During the year of probation she is moved from ward to ward, that she may gain a knowledge of medical as well as of surgical nursing; and in many hospitals lectures and practical demonstrations are occasionally given the probationers by the medical officers as well as by the matrons. On the expiry of her three years' engagement, whether the nurse elects to leave the hospital or not, she is entitled to a certificate if she has performed her duties satisfactorily, which certificate ought to be, and is usually, signed by representatives of the lay and medical authorities of the hospital and by the matron. One would suppose that a document of this nature would obviate the necessity of a public register, and no doubt it does to the initiated, but these, unfortunately, form but a small minority of the general public, and in too many instances, where the term certificated is employed, the claimant makes use of testimony of a very different complexion. Over and over again I have known discarded nurses fill offices of trust and responsibility on the strength of their hospital training, supported by the testimony of some kind friends they have made in the hospital, who were either ignorant of their shortcomings, or were disposed to look upon them with indulgent forbearance. Now a nurse possessing testimonials of this character, speaking literally, has a certain claim to call herself both hospital-trained and certificated. We have abundant proof that many are not slow to take advantage of the claim, but the fact discloses certain informalities in our present arrangements calling for correction. Nor am I by any means sure that the official certificate or diploma to which I have referred (although approaching the requirements of the proposed register) always conveys evidence of a similar and conclusive character as to the limit of time the nurse has been engaged in acquiring a knowledge of her profession. It is certain, at all events, that in some hospitals, chiefly provincial, nurses are certified as qualified to tend the sick after one year of hospital training. We are all, I think, agreed that this is too short a time to test the qualifications of a nurse.

Having regard to the fact that some diversity still exists in some hospitals with respect to the training curriculum, it is highly desirable that whatever arrangements are now in force or may in future be contemplated, should be controlled by a central authority to see that no one is entered on the register, whose character and antecedent qualifications are not up the mark. It is sufficiently clear also that the first duty of this authority must be to preserve a certain autonomy in the various training schools throughout the country with regard to the requirements of the register, and to fix with this object a minimum curriculum.

Although personally disposed to make the register, in the first instance at all events, as elastic as possible, I feel very strongly the necessity of restricting it to those only who have had hospital training and experience in medical and surgical nursing. Should specialists protest against their exclusion, my reply would be that the register was never intended for them, unless in the first instance they had undergone a suitable course of experience in medical and surgical nursing, and if the evidence is forthcoming there is no reason why the special qualification should not be annexed to their names on the register. On the above grounds I would be inclined to enter my veto against the names of midwives, monthly nurses, lunacy attendants, masseuses, and others being entered, unless they also possess the double qualification.

Now, with respect to the all-important question as to the time which ought to elapse in the course of her career before a nurse should be entitled to place her name on the register, or, in other words, the length of the curriculum. It may help us in part to solve the question if we notify the requirements of the various London training schools before a nurse can lay claim to her certificate, for it is clear that no nurse's name can appear on the register until her preliminary engagement has expired, and she is able to produce satisfactory proof of her competence. Let us see what are the regulations as to time at the hospitals associated specially with nursing institutions. The London Hospital, two years; St. Bartholomew's, three years; Guy's, three years; Westminster, three years; Middlesex, three years; University College, three years; Charing Cross, three years; St. Mary's, two years; Royal Free Hospital, two years; St. George's, three years; Homœopathic Hospital, three years. St. Thomas's is not included, as the nursing under the Nightingale Fund retains its connexion with the parent institution for an indefinite period, although nurses are sent from the hospital on various missions after one year's probation. It may be noticed that by almost general consent the time of probation and supervision extends over three years, the main exception to the rule being in the case of the London Hospital, where it is limited to two years. Miss Lückes however, informs me that in the event of nurses leaving the hospital for other work at the expiration of the period agreed on, she still retains a certain hold on them on account of her recommendation enabling them to fill similar offices either in hospitals, infirmaries, sick asylums, or nursing institutions. Possibly it may be found necessary to apply a modified rule to nurses who serve only one year in the parent hospital and two or more years in institutions co-operating with it; provided always that the nurse at the end of the curriculum can show evidence that her work has been performed satisfactorily.

You may gather from my remarks that I consider no one should be entitled to place her name on the register unless she can give satisfactory proof of having been at least three years engaged in nursing the sick, one year or more of which she has spent in a public hospital and the rest of the time in an institution or institutions more or less co-operating with the hospital. Further, that unless she can produce evidence from her employers that she is thoroughly acquainted with medical and surgical nursing, and is in every respect trustworthy, she ought not to be considered eligible for enrolment on the register. With respect to our present race of sisters and nurses who have served us faithfully, many far beyond the alloted term of three years, I would be disposed to recommend that they should be placed on the register *en bloc*, their claim to the distinction being indisputable. Under whatever auspices the register is established, as established it must be at no distant date, it will probably be found necessary, as is the case in all similar organisations, to require each candidate for registration to undergo some kind of examination, practical or otherwise, to test her capacity for nursing. This would be in addition to the certificate of the training school, but I have no desire to press this rather formidable matter, and anticipate events which must be left to the discretion and foresight of those to whom the registration is entrusted. The subject, however, leads naturally to the all-important question, With whom are we to register? Now, if a register for nurses is to be established at all, it must derive its authority from, and be under the protection of, some well-organised centre, possessing the confidence of the State, the various nurse-training schools, the medical profession, and it would follow, as a matter of course, the general public. Although I am reading this paper at the instance of The Hospitals Association, I am not prepared to say that it is the very best authority to delegate with such a grave responsibility. The Association is still young; it does its best to secure the good opinion of hospital authorities in London and the provinces, and is to a certain extent successful; but it is a voluntary society, lacking legislative power, and all those helps which a body constituted for a definite purpose has at its command. The most important object for which the General Medical Council for the United Kingdom was called into being was the education and registration of duly qualified members of the medical profession, and it has occurred to me as well as to others, that although the Medical Council may possibly be averse to taking up any subject outside its original commission, there is none in which the members or their constituents can have a deeper interest than in the protection and well-being of their immediate helpmates in the treatment of the sick. Time presses, the subject has been too long in abeyance, and the honest and industrious worker suffers by competition with the novice and the charlatan.

The expediency of the registration being conceded, we must not close our eyes to the fact that, apart from its inception, there are numerous difficulties that we will have to contend with in its future application. My experience has taught me that nurses as a rule are a very nomadic race, a fact attributable no doubt to the nature of their vocation, although something must be allowed for an inherent desire for change which possesses a great number. After servnig for a period in one institution they migrate to another, and another, and ultimately disappear, whether into private life or private practice it is difficult to say. I don't doubt that any difficulty that may arise from their removal from place to place may be easily obviated by their keeping the registrar fully informed of their whereabouts, but I am not so sure of another contingency, constantly embarrassing us in our dealings with nurses. One could hardly gather from Mr. Burdett's interesting exposition of the basis on which a provident superannuity fund should be framed for nurses, that he allowed sufficiently for the common accident of marriage among them. I regret I have no very reliable statistics of a special character to guide us on this point, and that I must fall back on the returns of the last census for England and Wales, showing the probabilities of marriage between the sexes. Well, the Registrar-General informs us that between the ages of twenty-five and fifty-five, a fraction over four-fifths, or eight out of ten, of the women living are either married or widows, and we have no reason to suppose that nurses go to swell the minority. On the contrary, as there is a very general presumption that they make the best wives and mothers, their chances of matrimony, I should say, were considerably above the average, raising it probably ten per cent. higher, and thus leaving only one-tenth of their number unmarried at the age of fifty-five. Are nurses to be excommunicated from the register because they get married? I think it would be very hard on them to suggest such a course, unless they desire it. Lady doctors do not appear to give up practice when they get married, and nurses have a much stronger claim on us than lady doctors.

But a register for hospital-trained and certificated nurses can hardly be accomplished in a day. It took many years before a similar register was obtained for qualified practitioners in medicine, and there are many aspects pertaining to this question which would be all the better by being thought over and discussed. I believe we have now from twenty to thirty nurse-training schools in the country, chiefly attached to large hospitals, in which women are systematically trained. I do not despair of bringing these separate establishments to a common understanding as to the qualifications and curriculum desirable before a nurse can be entrusted with a nurse's responsibility, and when we have done this we can go to the Medical Council, the Privy Council, or any other authority having the power of obtaining for us legal sanction for the register.

Her Majesty the Queen has expressed a wish that the money presented to her by the women of England as a jubilee gift should be used for the benefit of nurses and nursing institutions. It would be difficult to conceive a more appropriate object, but when the question is asked who are the nurses and which are the institutions entitled to benefit by Her Majesty's bounty, the almoners, in the absence of a register or any system of nurse organisation, will find its solution an almost impossible task. The same difficulty, I apprehend, will crop up at all attempts to form pension funds and other measures in contemplation for the benefit of nurses, and I am not at all sure that the committee of distribution, whoever they may be, could confer a greater boon on the whole nursing profession than by their obtaining for themselves legal authority to establish the register.

